# Bromodomain Inhibitor Review: Bromodomain and Extra-terminal Family Protein Inhibitors as a Potential New Therapy in Central Nervous System Tumors

**DOI:** 10.7759/cureus.620

**Published:** 2016-05-21

**Authors:** Elizabeth Wadhwa, Theodore Nicolaides

**Affiliations:** 1 Pediatrics, University of California, San Francisco; 2 Neurological Surgery and Pediatrics, University of California, San Francisco

**Keywords:** brain tumors, neuro-oncology, bromodomain inhibitors, pediatrics

## Abstract

The bromodomain and extraterminal (BET) family proteins associate with transcriptional activation through interaction with acetylated chromatin, therefore playing a key role as epigenetic regulators. BET proteins serve to regulate the expression of importance oncogenes, including those involved in apoptosis as well as cell cycle progression. Due to this potential as an epigenetic target, small molecule inhibition of BET proteins have been investigated and demonstrate promising activity in both solid and hematologic malignancies, including brain tumors. Glioblastoma multiforme (GBM), subsets of medulloblastoma, and diffuse intrinsic pontine glioma (DIPG) are types of brain tumors with dismal prognoses, and as such have been the subjects of preclinical studies using BET inhibitors both *in vivo* and *in vitro*. While results from these preclinical investigations have shown promise, clinical trials are in early phases at this time. In this review, we will summarize the current literature on BET family proteins, their potential as therapeutic targets in brain tumors as well as other malignancies, and the preclinical and clinical investigations that have been undertaken to date.

## Introduction and background

### Role of bromodomain proteins in cells

The lysine acetylation on the N-terminals tails of histones is associated with activation of transcription through opening of chromatin architecture [1]. This histone acetylation allows for transcriptional complex assembly by recruiting bromodomains [2]. “Bromodomain” is often the term used to describe bromodomain and extra-terminal (BET) family of human bromodomain proteins (BRD2, BRD3, BRD4), which share the structure of two evolutionarily highly conserved N-terminal bromodomains as well as a C-terminal domain with potential for more divergence in sequence [3-4]. Bromodomains, which are proteins with acetyl-lysine binding modules, function as the principal mediators of molecular recognition of acetylated chromatin and therefore have a key role in transcriptional activation [5-6]. Interestingly, BRD4 bromodomains have been shown to bind propionylated and butyrylated lysine residues [7]. They not only serve in targeting of chromatin-modifying enzymes to specific sites, but also interact with other protein modules (such as PTEFb, Rb, E2F and the SWI/SNF complex) in order to increase targeting specificity for these enzymes [8]. Bromodomains have been identified in nuclear proteins including transcriptional coactivators, histone acetyltransferases, methyltransferases, and chromatin-remodeling complexes [9-12].

### BET family proteins as targets in cancer cells

In the past 10 years, research has established a compelling rationale for targeting BET proteins for their oncogenic potential. BRD4 influences mitotic progression by binding to transcriptional start sites of genes and directing post-mitotic transcription [13]. BRD4 also mediates transcriptional elongation and has been linked to c-MYC dependent transcription as well as positive transcription elongation factor complex (P-TEFb)-induced increased growth promotion [14-15]. The discovery of the roles of BRD4 and the other proteins in the bromodomain family established a rationale for targeting BET bromodomains to inhibit transcription, particularly in cancer cells. 

Epigenetic proteins such as BET have long been considered potential therapeutic targets and as such investigators had pursed them in ligand discovery. The oncogenic potential of BET proteins was first identified in nuclear protein in testis (NUT) midline carcinoma, a highly malignant solid tumor in which oncogenesis is driven by fusion of BRD3 or BRD4 with the NUT protein.This fusion results in blockade of epithelial differentiation and promotion of tumor growth [16]. This discovery prompted initiation of testing potential BET inhibitors in this cancer while expanding to test for BET activity in other malignancies. 

### Current BET inhibitors

Filippakopoulos et al. were the first to describe a histone binding module inhibitor. They reported JQ1, a cell-permeable small molecule that competitively binds to bromodomains with high potency and specificity. When JQ1 binds, it displaces the BRD4 fusion oncoprotein from the chromatin, thus inducing cell-cycle arrest and initiating apoptosis. The discovery of the first BET inhibitor by this group led to the development of other BET inhibitors that probe throughout the BET family [3]. 

Other BET inhibitors include I-BET151/762, PF-1, and RVX-208. Long et al. used a Hedgehog-dependent report cell line (Light2 cells) to target different epigenetic modulators to find that I-BET151, a BET protein inhibitor attenuates Hedgehog activity. As therapy with I-BET151 mimicked BRD4 knockdown, their data suggests that BRD4 regulates the Hedgehog signaling pathway [17]. I-BET762 is a similar synthetic compound that works by mimicking acetylated histones to disrupt chromatin complexes [18]. PF-1 is a potent and highly selective dihydroquinazoline-2-one inhibitor, which blocks the interaction of BET bromodomains with acetylated histone tails. Picaud et al. used cocrystal structures to show that PFI-1 acts as an acetyl-lysine (Kac) mimetic inhibitor by efficiently occupying the acetyl-lysine binding site in BRD2 and BRD4. PF-1 results in arrest of the cell cycle in G1, downregulation of MYC and Aurora B kinase, and apoptosis induction [19]. RVX-208 is a BET inhibitor with preferred binding to the second bromodomains (BD2s), with highest selectivity observed for BD2 of BRD2 and BRD3. While the compound is currently being investigated in phase II clinical trials for cardiovascular diseases, Picaud et al. (2013) demonstrates the feasibility of specific targeting within the BET family resulting in different transcriptional outcomes [20]. Current inhibitors include BMS-986158, OTX015, and PLX-51107, among others in development; the mechanism of action of each of these inhibitors is slightly varied.

### Preclinical studies of BET inhibitors

Over the past few years, these newly described small molecule BET inhibitors have been tested *in vivo* and *in vitro* and shown to have potent antiproliferative effects not only of NUT (nuclear protein in testis)-midline carcinoma, but also hematologic malignancies, including leukemia and multiple myeloma, and other solid tumors, including lung, thyroid, liver, colorectal, prostate, and skin cancers, neuroblastoma, sarcoma, and brain tumors [21-34].

Pediatric malignancies for which BET inhibition has been investigated for therapeutic potential include acute lymphoblastic leukemia (ALL) and neuroblastoma. Pediatric B-precursor ALL is the most common childhood cancer and in most cases a highly curable disease. However, the treatment resistance and long-term toxic side effects of current therapies in a subset of patients pose the need for more targeted therapies, prompting the consideration of BET inhibition as a potential therapeutic approach [35]. Ott et al. showed JQ1 potently reduced the viability of those B-ALL cell lines with high-risk cytogenetics, particularly lines with CRLF2 rearrangements. They found JQ1 downregulated transcription of IL7R, which normally heterodimerizes with CRLF2 and signals through JAK1/2 and STAT5 pathways to promote cell proliferation. JQ1 was also shown to reduce JAK2 and STAT5 phosphorylation and deplete BRD4 from the IL7R promoter. In xenograft studies with CRLF2-rearragned B-ALL, JQ1 suppressed MYC expression and STAT5 phosphorylation, prolonging survival [32]. Da Costa et al. showed a potent *in vitro* cytotoxic response to JQ1 in a panel of primary ALL cells. This response was independent of prognostic features but did depend on high expression of MYC and coupled with transcriptional downregulation of various pro-survival pathways. JQ1 decreased c-MYC protein stability and also reduced progression of DNA replication forks. JQ1 sensitized the ALL cells to dexamethasone therapy [36].

Neuroblastoma is the most common extracranial solid tumor in childhood. While children diagnosed at younger ages and earlier stages tend to have favorable prognosis, this diagnosis continues to carry a dismal prognosis for those diagnosed with advanced stage or relapsed disease. Many of the high-risk neuroblastoma cells are MYCN-amplified; therefore novel therapeutic strategies directed toward this target are continually being studied. Puissant et al. conducted a cell-based screen of genetically defined cancer cell lines using a prototypical BET bromodomain inhibitor to reveal a robust correlation between MYCN amplification and sensitivity to bromodomain inhibition. Neuroblastoma is frequently associated with amplification of MYCN, and bromodomain-mediated inhibition of MYCN attenuated growth and induced apoptosis, conferring a survival advantage in three *in vivo *models of neuroblastoma [37]. Lee et al. validated the antitumorigenic effects of JQ1 in neuroblastoma using monolayer and sphere-forming conditions *in vitro* and subcutaneous neuroblastoma xenografts *in vitro*. They also looked at neurofilament expression to assess whether JQ1 induced neural differentiation. They found BET inhibition to attenuate progression and promote neuronal differentiation of neuroblastoma *in vitro* and *in vivo* in mice [27]. 

## Review

### Preclinical studies of BET inhibitors in brain tumors

Extensive preclinical work has been performed to determine the potential efficacy of BET in brain tumors. Glioblastoma multiforme (GBM), the most common and aggressive primary malignant brain tumor, bears a dismal prognosis and therefore presents a challenge for development of novel therapeutic strategy. In considering epigenetic proteins and their recent emergence as novel anticancer targets, several studies have looked at BET proteins as potential targets for therapy. 

One BET inhibitor that has been studied with GBM is JQ1. Cheng et al. assessed JQ1 in a panel of genetically heterogeneous GBM samples. They used ex vivo cultures derived from primary GBM xenograft lines and orthotopic GBM tumors to test *in vivo* efficacy. They found that JQ1 induced marked G1 cell-cycle arrest and apoptosis, resulted in significant changes in the expression of important GBM genes, including c-MYC, p21CIP1/WAF1, hTERT, Bcl- 2, and Bcl-xL. They also found that the efficacy of JQ1 was not compromised by Akt hyperactivation or p53/Rb inactivation, indicated that these often-mutated signaling pathways may not confer resistance to JQ1. The orthotopic GBM tumors also showed significant growth repression with JQ1. The results of these studies support the potential broad therapeutic use of BET bromodomain inhibitors in the treatment of GBM tumors [23]. Liu et al. used integrated epigenome and transcriptome analyses of cell lines, genotyped clinical samples, and The Cancer Genome Analysis data, to show that EGFR mutations remodel the activated enhancer background of GBM to promote aggressive tumor growth through a SOX9 and FOXG1-dependent transcriptional regulatory network *in vitro* and *in vivo*. They found that EGFRvIII, the most common EGFR mutation, sensitizes GBM cells to the BET-bromodomain inhibitor JQ1 in this SOX9, FOXG1-dependent manner, thereby identifying the role of epigenetic and transcriptional remodeling in EGFR-dependent tumorigenesis and suggesting a plausible basis for epigenetic therapy [38]. 

Another inhibitor that has been evaluated in terms of efficacy in GBM tumors is I-BET 151. Pastori et al. (2014) perfomed NanoString analysis of GBM tumor samples to demonstrate the significant overexpression of BRD2 and BRD4 RNA in GBM as well as the reduced cell cycle progression with disruption of BRD4 expression. They also used cell proliferation assays of GBM cell lines and stem cells and performed xenograft experiments to determine the efficacy of I-BET151, a BET protein inhibitor. Their studies showed that treatment with I-BET151 arrests cell cycle progression, reducing GBM cell proliferation *in vitro* and *in vivo* [39]. To understand the mechanism through which BET protein inhibition reduces GBM growth, Pastori et al. (2015) used single molecule sequencing to identify a subset of GBM-specific long noncoding RNAs (lncRNA) whose expression is regulated by BET proteins. They found that treatment of GBM cells with I-BET151 reduced levels of the tumor-promoting lncRNA HOX transcript antisense RNA (HOTAIR), subsequently restoring expression of other GBM downregulated lncRNAs. Their findings conversely included that overexpression of HOTAIR in conjunction with I-BET151 therapy nullifies the antiproliferative activity of the inhibitor. Their findings suggest that modulation of lncRNA networks may partially mediate the antiproliferative effects of many epigenetic inhibitors [40]. The results of all these studies support the potential broad therapeutic use of BET bromodomain inhibitors in the treatment of GBM tumors.

Medulloblastoma is the most common malignant brain tumor in children. The current consensus is of at least four distinct subtypes, including Wingless (WNT), Sonic Hedgehog (SHH), and groups 3 and 4 [41, 42]. Amplifications of MYC, MYCN, or MYCL are found in several subtypes, including group 3 tumors, which have the worst prognosis, as well as some SHH and group 4 tumors [43, 44]. Patients with medulloblastoma tumors that overexpress MYC or harbor a MYC oncogene amplification have an extremely poor prognosis. The MYC protein has been a notoriously difficult direct target for novel drug development, and BET bromodomain inhibition is a potential therapeutic strategy to target c-MYC [2, 45]. Henssen et al. evaluated the efficacy of JQ1 against preclinical MYC-driven medulloblastoma models. They found treatment with JQ1 to significantly reduce cell proliferation and preferentially induce apoptosis in these cells by downregulating MYC expression and therefore resulting in transcriptional deregulation of MYC targets; their results showed significantly prolonged survival and reduced tumor burden in mice xenografts [46]. Bandopadhayay et al. used newly generated patient and genetically engineered mouse model-derived medulloblastoma cell lines and xenografts with MYC or MYCN amplifications to find that JQ1 suppresses MYC expression, MYC-associated transcriptional activity, and therefore decreased cell viability in medulloblastomas [44]. Venkataraman et al. also showed that bromodomain inhibition of medulloblastoma with JQ1 restricts c-MYC driven transcription, suppresses cell growth, induces apoptosis, and suppresses stem cell-associated signaling, inhibiting self-renewal of medulloblastoma tumor cells. Additionally, they found that JQ1 also promotes cellular senescence through activation of cell cycle kinase inhibitors and inhibition of E2F1 activity, an effect that was also observed *in vivo *[47]*. *The preclinical data in these studies supports the pursuit of testing BET inhibitors as molecular targeted therapeutic options for patients with MYC-driven medulloblastoma.

Medulloblastoma has other potential therapeutic targets for non-MYC-driven tumors. Tang et al. first described the use of BET inhibitors in Hedgehog-driven cancers, including medulloblastoma. They found medulloblastoma as well as atypical teratoid/rhabdoid tumors (ATRT), another pediatric brain tumor with non-canonical Hedgehog signaling, to respond to treatment with BET inhibitor JQ1 [48]. As described above, Long et al. found BET protein inhibitor I-BET 151 to result in decreased Hedgehog activity. They found that I-BET151 suppressed the Hedgehog activity-dependent growth of medulloblastoma cells, both *in vitro *and *in vivo* [17].

Diffuse intrinsic pontine glioma (DIPG), the most common brainstem tumor of childhood, is almost uniformly fatal, and current treatment options provide very little survival advantage. The high level of MYCN in diffuse intrinsic pontine glioma (DIPG) led investigators to test sequential therapy with the bromodomain inhibitor JQ1, which targets MYCN, and the gamma-secretase inhibitor MRK003, which targets NOTCH. Taylor, et al., found that this dual targeting with JQ1 and MRK003 inhibited DIPG growth and induced apoptosis, suggesting this as a potentially effective therapeutic regimen [49].

### Current clinical trials of bromodomain inhibitors in brain tumors

With the promising results of preclinical trials, there are currently several open trials with different BET inhibitors that are actively recruiting participants with various hematologic and solid malignancies. Several of these trials are open or have recruited solid tumors of all types, including brain tumors, and each of them tests a different BET inhibitor with slight variation in mechanism. JQ1 is not being tested in clinical trials due to its short half life. Open trials include a GlaxoSmithKline-sponsored phase I/II study of GSK525762 in subjects with NUT midline carcinoma and other cancers, an Incyte Corporation-sponsored phase I/II of INCB054329 in subjects with advanced malignancies, and Tensha Therapeutics phase I, multicenter, open-label study of TEN-010 for patients with advanced solid tumors. While neither of the following studies is actively recruiting, GlaxoSmithKline has a dose escalation study of GSK2820151 in subjects with advanced or recurrent solid tumors, and Bayer has a phase I study of BAY 1238097 for subjects with advanced malignancies. The only trial in which a BET inhibitor has specifically been tested in brain tumors is a phase IIa trial of OTX015, which tested dose optimization in recurrent GBM patients. This was a non-randomized, multicenter study of single agent use of the inhibitor following failure of front-line therapy; the trial was terminated after one year. There are no current open trials with BET inhibitors in pediatric brain tumors.

## Conclusions

It is now apparent that BET family proteins play a critical role in transcriptional activation and have oncogenic potential. This discovery that BET proteins can act as potential therapeutic targets in cancer has led to the development of several small molecule BET protein inhibitors. Preclinical studies have shown promise in a number of hematologic and solid malignancies both *in vivo* and *in vitro*. There are several open early phase clinical trials that are currently testing the efficacy of these inhibitors in humans. Brain tumors, including GBM, DIPG, and subsets of medulloblastoma, bear dismal prognoses with current therapies, and while BET protein inhibitors have been positively tested in preclinical trials with these pathologies, clinical trials currently are in early stages. Future directions include more extensive preclinical trials of brain tumors with various BET protein inhibitors as they continue to be developed, as well as translation of promising results to phase I/II clinical trials.
